# Identifying the needs of women following a severe postpartum hemorrhage

**DOI:** 10.18332/ejm/183027

**Published:** 2024-06-29

**Authors:** Imelda Fitzgerald, Joye McKernan, Richard Greene, Rhona O'Connell

**Affiliations:** 1 Cork University Maternity Hospital, Cork, Ireland; 2 National Epidemiology Centre of Ireland, University College Cork, Cork, Ireland; 3 School of Nursing and Midwifery, University College Cork, Cork, Ireland

**Keywords:** postpartum hemorrhage, informational, emotional, support, midwives, care-pathway

## Abstract

**INTRODUCTION:**

Childbirth is a unique experience for women. In Ireland, major obstetric hemorrhage (MOH) is the most frequently reported severe maternal morbidity (SMM) with an incidence of 3.27 per 1000 maternities. Much is known now about the management of postpartum hemorrhage (PPH), and there is some research on women and their partner's experience. Less is known about how the woman feels emotionally following a PPH or what informational needs and emotional support are required. The aim of this study was to understand how women felt after experiencing a severe PPH, to listen to their first-hand experience, and to learn what improvements could be made for future care for women who experience a PPH.

**METHODS:**

A descriptive, quantitative approach was conducted using semi-structured interviews with women who had a severe hemorrhage (blood loss) of ≥2.5 L between four and fourteen months postpartum.

**RESULTS:**

Five women took part in this study. The women identified a lack of information provided to them about the reason for the significant bleeding. The women voiced they could overhear information about the event discussed between healthcare professionals but not with the woman. The care the women received in the High Dependency Unit (HDU) was significantly different from the care they received in the postnatal wards, and the women were not informed they were clinically well for transfer to the postnatal ward. It was reported that the postnatal wards were busy and short-staffed, and the women looked for more emotional support from staff, which was not available. This had an effect on their recovery in the postnatal period.

**CONCLUSIONS:**

The women reported that they wanted more information in the early postnatal period following the event, and some still had unanswered questions at the time of the interviews several months later. Most of the participants did not receive adequate emotional support from the midwives caring for them, which resulted in the participants requesting early discharge home to get emotional support from members of their family.

## INTRODUCTION

In Ireland, major obstetric hemorrhage (MOH) is the most frequently reported severe maternal morbidity (SMM) with an incidence of 3.27 per 1000 maternities^1^. Postpartum hemorrhage (PPH) is defined as excessive vaginal bleeding following birth. It may be from the uterus, cervix, vagina, and perineum, over 500 mL, while a severe PPH is defined as a blood loss of over 1500 mL within a 24-hour period^2^. A severe PPH is an obstetric emergency that needs immediate intervention and is a leading cause of maternal death^3^. Globally, the rate of PPH has been increasing. In the Republic of Ireland, a MOH (blood loss ≥2500 mL) is the leading cause of Severe Maternal Morbidity (SMM) in 55% of all cases^4^. SMM are unintended outcomes of the process of labor and birth that result in significant short-term or long-term consequences to a woman’s health^1^.

In 2015, there were 7894 births, with 433 (5.4%) women who experienced a primary PPH and 2.7% (n=213) having a severe PPH. The rate of MOH has increased in Ireland by 43% in the last decade. For women with severe PPH, there is a risk of invasive treatment (bimanual compression, balloon tamponade, hysterectomy)^5^, which can exacerbate psychological trauma (anxiety, depression, PTSD)^6^.

Within the last decade, studies from the UK, Australia, New Zealand, and Denmark have reported on the needs of women after different volumes of blood loss^7-9^. The majority of research focuses on the management rather than how women may be affected by a bleed^10^, but reports on women’s experience of PPH following childbirth identified that women have informational, physical, and emotional needs in the short- and long-term^11-14^.

Part of the role of a midwife is to advocate for the woman to ensure her safety and make sure they have all the information they need to make informed choices about their care^15^. Women’s experience of PPH has not been explored in the Irish maternity services, so the aim of this study was to identify the support needs and concerns of women subsequent to the severe PPH.

## METHODS

### Design and setting

This qualitative, descriptive research, using semi-structured interviews, was carried out across a tertiary maternity hospital in the south of Ireland, which can provide care for critically ill women and newborns. All women following a moderate and severe PPH are admitted to the HDU for monitoring and treatment. Depending on acuity, a woman may receive care in the maternity HDU or transfer to a general ICU if required. In Ireland, every pregnant person is entitled to free maternity care for herself and her baby.

Within this unit, the management of PPH adheres to the national clinical guideline on PPH^2^, which includes assembling a multi-disciplinary team to treat and manage the event. In the case of a PPH ≥1000 mL and ongoing bleeding or retained placenta, the woman is transferred to the operating theatre to control the blood loss. Depending on the severity of the hemorrhage, the woman is transferred to the HDU for monitoring and, once clinically stable, transferred to the postnatal ward.

Ethical approval for the study was provided by University College Cork (UCC) Cork Research Ethics Committee (CREC) ECM 4(bb) 04/04/17), and permission was also provided by the local hospital management committee to undertake the research.

### Recruitment

The HDU admission record book was reviewed for women who were eligible to participate. Between December 2015 and April 2017, there were 30 women admitted to the HDU following a PPH of any volume. Twelve of the women experienced a PPH of <2.5 L, five infants were admitted to the Neonatal Unit (NNU), four women had an antepartum hemorrhage (APH), one woman lived abroad, one woman did not speak English as a first language, and one woman was under the age of 18 years.

Once the eligible criteria were applied (Table 1), there were six women suitable to contact for the study. These women were contacted by phone about the project, and if interested, a participation leaflet was sent in the post with contact details for the study. A briefing telephone call was arranged to discuss the study, and if the participant was happy to proceed, a suitable time and date were arranged. Of the six eligible women, one declined participation.

**Table 1:** Inclusion and exclusion criteria of women who had a severe hemorrhage between four and fourteen months postpartum, across a tertiary maternity hospital in the south of Ireland (N=5)

*Inclusion criteria*	*Exclusion criteria*
PPH ≥2.5 L Admitted to HDU Spoke English Aged >18 years PPH within 18 months	Infant transferred to NNU Antepartum hemorrhage (APH) Fetal abnormalities Secondary PPH Hysterectomy

### Data collection

The data were collected using semi-structured face-to-face interviews as this can give the participant the opportunity to express their point of view and may result in understanding and learning from their perspective^16^. One author carried out all the interviews. Prior to each interview, the purpose of the study was explained, and the participant could withdraw at any time. Arrangements were made that if any woman was distressed by the interview or if she had unmet needs, she could have an appointment to meet an obstetrician involved in her care. Two participants voiced that they had unanswered questions relating to the event. Both were offered a referral to the maternity hospital; one woman was referred to an obstetric consultant for review, and the second woman wanted information on how to be referred in case she decided to proceed with a review in the future.

Participants were informed that all data would be confidential, and pseudonyms would be used for transcription and storage of the data. If the participant was happy to proceed, a consent form was signed. The women were encouraged to speak freely and include any aspects of their care so that improvements could be made. Interviews took place in the women’s home or at their place of work as two of the participants made this request. They lasted 45–60 minutes, and all data were recorded and transcribed before coding and analysis commenced.

### Interview tool

A previously used interview schedule by Dunning et al.^7^ was adapted, as the topic areas were similar and the findings could be compared. Permission was granted to use and amend the tool. The original interview schedule comprised fifteen open-ended questions. Two of the original questions were removed as not relevant to this study, and other questions were added to reflect local service provision. It became a sixteen-piece open-ended interview schedule (Supplementary file).

### Data analysis

All interviews were recorded and then transcribed verbatim using pseudonyms for all names. The transcribed text was analyzed using Braun and Clarke’s^17^ thematic analysis to identify patterns in the data. This consisted of six steps:

1. *Becoming familiar with the data:* Following each interview, the interviewer listened to the audio recording, and each transcript was read several times in order to make comparisons between the interviews and to develop a deeper understanding of the participants’ perspectives. The interviews and findings were reviewed and discussed with the other members of the team.
2. *Generating codes:* The authors began by identifying topics in the raw data sets as code. In this data set, several codes were developed; following discussions and review, the agreed codes were named: experiences, supports, knowledge, and improvements. This process allowed the authors to focus on the distinctive themes that emerged in the data.
3. *Generating themes:* Each of the transcripts were compared to identify common experiences, care, or issues reported. The themes that were found were: a common experience, lack of information, debrief, and enhancements.
4. *Reviewing themes:* The researchers independently reviewed the coding and the themes that were found until data saturation was achieved. There was an overlap between two of the original themes: lack of information, and debrief. These themes were found to have similarities, and a decision was made to incorporate them into one theme.
5. *Defining and naming themes:* The researchers wanted to ensure the themes reflected the story of the data, and through several meetings, there were four themes named: reflections of the event, information gaps, perception of staff, and enhance future care.
6. *Locating exemplars:* For each theme, several quotations from the data are included to share the experience of the women in this study.

**Figure 1:**
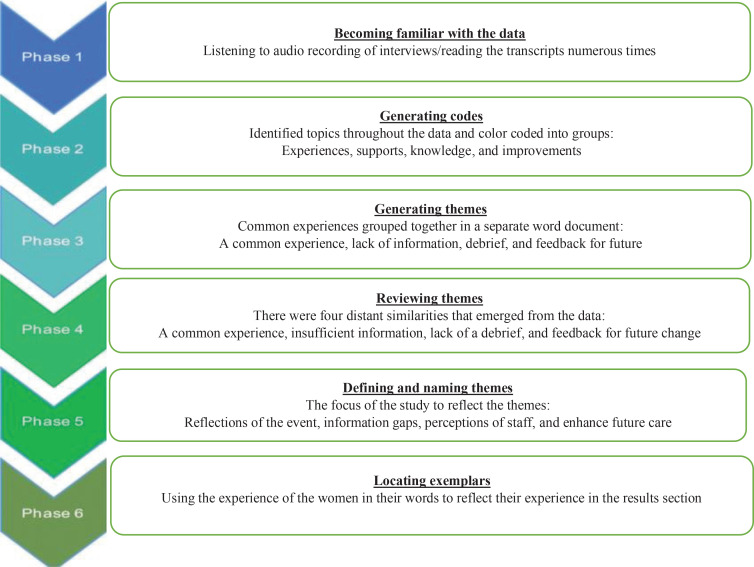
Summary of the six phases of thematic analysis

Figure 1 illustrates the steps taken during the analysis.

## RESULTS

The majority of the women were primiparous and breastfed their infants. The blood loss volume ranged 2.5–3.0 L (Table 2).

**Table 2:** Maternal demographics of women who had a severe hemorrhage between four and fourteen months postpartum, across a tertiary maternity hospital in the south of Ireland (N=5)

*Pseudonym*	*Age*	*Parity*	*Mode of birth*	*Augmentation*	*Volume of blood loss (L)*	*Feeding method*	*Age of the infant at the time of the interview (months)*
Sarah	29	1	Elective cesarean	No	3.0	Breastfeeding	14
Veronica	27	2	Spontaneous vaginal delivery	Yes	2.5	Breastfeeding	11
Amy	33	1	Instrumental	Yes	3.0	Breastfeeding	6
Sally	31	1	Emergency cesarean	Yes	2.5	Artificially feeding	7
Clare	26	1	Instrumental	Yes	2.5	Breastfeeding	4

Each of the women recalled their experience of the first few hours and days following the PPH. The participants highlighted how the lack of information and how feeling unsupported impacted them. There were suggestions made on potential improvements for other women who experience PPH.

The themes that were developed and represented core descriptions among participants were: 1) Reflections of the event, 2) Information gaps, 3) Perception of staff, and 4) Improvements for practice.

### Reflections of the event

This reflects on the PPH and the women’s experience of being in the HDU. Though the interviews were between 4 and 14 months postpartum, each woman recalled vivid details about what had occurred. Fear, pain, and confusion from the event were discussed.

The women described how it felt when an influx of healthcare professionals entered the birthing room and were discussing care without involving the women:

*‘I didn’t know the difference between the scrubs.’* (Amy)

The experience was traumatic for the women as none of them expected this to happen after the birth:

*‘It was a very frightening experience, where I had to distance myself from what was happening and tell myself, “It’s ok, it’s ok, they know what they’re doing”.’* (Sally)

One woman recalled the severe pain experienced when invasive procedures were used:

*‘I was in a lot of pain when he had to do several internals; at one point, I nearly kicked the doctor as the pain was so bad.’* (Amy)

Another expressed a sense of panic when she woke up in the HDU after a cesarean birth. She spoke of how the fear she experienced did not allow her to take in information about what had happened or why she was there:

*‘I didn’t really realize that something had happened to me, and that was the reason I was in the HDU. I thought it was because of the emergency cesarean section. The information I needed to reassure me was slow to be given, it trickled in from hearing the midwives’ handover to each other, those little bits of information they were like “little snippets”.’* (Clare)

Each of the women discussed the transition from the individualized care they received in the HDU from a midwife to the differences in care level once admitted to the postnatal ward:

*‘It was a pretty scary when I got up there and realized there’s no one here to help me, it would be just her [the baby] and me for the first time.’* (Veronia)

*‘I was still so weak when I went upstairs [postnatal ward], I couldn’t walk or anything, and I was terrified of looking after her [the baby] on my own, I didn’t know what was going on, I needed my mum or my sister or my partner to help me day and night.’* (Sarah)

None of the women felt safe when they were transferred to the postnatal ward. They were often not informed that they were ‘clinically safe’, and they found the change of environment overwhelming. The women interviewed missed the one-to-one care received in the HDU and were in need of more support.

### Information gaps

Only one participant considered that she was given an adequate debrief following their PPH. She was informed of the treatment and the care she would receive while in the HDU. This provided her with reassurance to feel safe.

There were several occasions where the information provided was suboptimal. Two women were not aware they had a PPH. They thought they had been transferred to the HDU as a result of the cesarean section:

*‘The two bleeds were due to what was it called placental abruption, and then I had another one due to uterine atony, and the only reason I knew what that was, those words, my husband is a doctor, so he was able to explain that to me, the information was like not ideal in the way it was presented to me.’* (Clare)

One woman stated that she learned what had happened to her from overhearing the staff discussing her care. Only one woman felt she was given an adequate debrief, and her plan of care explained to her. Most women asked midwives on the postnatal ward for answers about the bleeding, but the postnatal staff were unable to provide the women with what they needed as they had limited information about the event:

*‘I asked, and the staff could only tell me what was on their sheets (notes), I don’t know if they were told about it, they explained what meconium grade two was. They didn’t talk about the bleed or anything about that; there wasn’t too much information.’* (Amy)

*‘I asked the staff on the ward what happened, and they read my notes and said I was so lucky. Until that point, I didn’t realize the seriousness of the bleed, and I questioned could I have died?’* (Veronica)

The women were provided with standard postnatal discharge information, but there was no specific information related to how to care for themselves at home following a PPH. The women looked for additional information on ways to heal physically and emotionally:

*‘I think they said to take iron, and around the bleeding, I think I asked what would be the amount to come back into hospital with, and they told me that, but that was it.’* (Sally)

*‘… if they had said you’re going to need a lot of support. You’re probably going to nap a lot, and you’re going to need someone to help you make food and assist you. You can forget about the washing – if something like that was said, it would have helped a lot.’* (Veronica)

The women wanted information about the emergency event. Often, this needs to be repeated, both when the woman is in the HDU but also on the postnatal ward before discharge.

### Perception of staff

The women commented that they needed more physical and emotional support from postnatal staff to care for their newborns. Most participants reported that the staff were friendly but that the midwives were too busy and did not get the additional support they needed:

*‘I was so weak; I couldn’t walk or do anything. I was terrified of looking after my baby on my own. I didn’t know what was going on; I needed my mum, sister, and partner to help me.’* (Sally)

*‘I would get out of the bed and bring her [baby] to bed to feed, and I didn’t know if I should be ringing the nurse [sic midwife] and the staff members. I didn’t know what was normal or what was, to a degree, worse because of the bleed.’* (Amy)

The midwives in the postnatal ward had several mothers and babies requiring care, often with complex needs:

*‘I would get out of the bed and bring her into bed to feed, and like, I didn’t know if I should have been ringing the midwife, but I didn’t know was that was normal or to a degree worse because of the bleed.’* (Clare)

Four of the five women breastfeed their babies:

*‘My milk didn’t come in until day 6, she was starving, and even then, it was, um, very slow coming in.’* (Veronica)

Sally wanted to be discharged home early as she felt she would have a better chance to be successful at breastfeeding at home rather than in the hospital:

*‘I slept a lot during the day and woke for her feeds, the only thing I did worry about was my tiredness, and at night when I fed her, I was just so worried that I would fall asleep.’* (Veronica)

An awareness of women’s experience of traumatic birth is important for midwives working on a postnatal ward to ensure that women get the support and information they require.

### Enhance future care

The women suggested future care should include that additional advice and support; Clare said if she had been given an information leaflet with ‘what to expect after a PPH’, it may have made a difference as the ‘not knowing and not trusting’ caused doubt:

*‘If there had been an informational booklet with practical ways to care for mum, baby, and partner, that would have been beneficial.’* (Clare)

Amy voiced that there were still unanswered questions about breastfeeding, diet, and follow-up. One of the recommendations made by the participants was the choice to have a visitor stay overnight to support the mother and newborn, which could have made a difference for the woman as they could have rested more.

## DISCUSSION

The results suggest that severe PPH may have short- and long-term complications if the woman’s physical, emotional, and informational needs are not met following a severe PPH. All women interviewed had unanswered questions regarding the PPH. The lack of emotional support resulted in the women feeling frightened; the areas of most concern were fear of being left alone, self-doubt, and fear regarding early discharge from the hospital. These findings were also reported by Thompson et al.^14^.

In this study, it was evident that there was a lack of information provided in a clear and timely manner for the women to understand the cause, management, and treatment of PPH. The follow-up care left the woman with the uncertainty of what to expect as normal experiences following a PPH; this was also found by Snowdon et al.^18^, where the women felt disempowered by the lack of communication between staff and the women.

Significant implications resulted for some of the women in the weeks and months following the severe bleeding and had an adverse impact on all the women’s opinions of their overall care. Rosvig et al.^9^ found that women wanted information about what to expect in terms of their physical recovery after a PPH and that it would have been helpful to know about what support services would be available.

The women wanted the midwives to spend time with them while they adjusted from the one-on-one care they received in HDU. This was also reported by Elmir et al.^11^, who found suboptimal postnatal care, a lack of breastfeeding support and emotional support after a severe bleed. In that study, some of the women could not trust the staff and depended on their families for support.

This study has been able to identify several similarities in the findings with published research^7-10^, and this reinforces that women who experience a PPH across the globe have overlapping feelings and emotions about the event and the care. There were also new findings; throughout this study, there was a lack of information provided to the women about PPH and how this impacted them individually in the subsequent months. It was identified that a care pathway is needed that has a standardized approach to ensuring the woman’s physical, informational, and emotional needs are included and provided by midwives and obstetricians in their daily postnatal care to women and following discharge. A care pathway could also assist midwives and obstetricians in their daily practice in meeting physical, informational, and emotional needs by having a checklist that would be tailored for each woman. Better communication and supportive strategies have previously been recommended to ensure that this vulnerable population of women is not disempowered by this traumatic birth experience^19^.

There were several suggestions from the participants to enhance future care. Suggestions included leaflets with how the women can care for themselves, what to expect, how partners can care for them, following a PPH, including nutritional needs.

The women also noted that the option for a visitor to stay overnight in individual cases for additional support should be explored. In Ireland, it has recently been recommended in the latest national guideline for the management of PPH that the woman and their birthing partner be offered a debrief as soon as possible after the event. A subsequent debrief should be offered to the woman and her partner following discharge home, usually around six weeks following childbirth^20^.

Though this study was small, we observed and reviewed the international research and noted that the findings are still relevant. Women following a postpartum hemorrhage are not getting optimum physical, informational, or emotional support in the short- and long-term. Postnatal wards are frequently understaffed, and women report more negative experiences of postnatal care where there are shortages of midwives^21^. The WHO Guidelines remind us that women should have a positive experience of their intrapartum care^22^ and also their postnatal care^23^. PPH levels have increased in recent years, and it is not always easy to resolve; some women experience significant blood loss before this can be adequately controlled. Care plans should be in place to ensure that these women are well supported throughout the event, but also that their postnatal and follow-up care should ensure that their needs are met to enhance their long-term health.

### Strengths and limitations

This study highlighted the woman’s experience and gave a platform for their voices to be heard. The findings from this study may also resonate with other women who may have experienced a traumatic birth. The first limitation of this study was the sample size, as there were five participants recruited. A larger sample size may have given a difference in experience and needs following a large bleed. The second limitation was the quantity of blood loss that the women needed to lose to be eligible for this study. Women who lost less blood volume did not have the opportunity to discuss what their needs were after a PPH; this means we are unable to compare if women who have minor or moderate bleeds have the same needs as women who experience a PPH of over 2.5 L.

## CONCLUSIONS

Each of the women had a different birth story, but they had similarities when it came to the emotional response and the impact of trauma, especially the issue of inadequate information. This may also be relevant for other women who experience other traumatic or unexpectedly complicated birth issues. Despite known risk factors for PPH and the current management and treatment of PPH, the rates of PPH are increasing globally. There is a need to ensure women who experience PPH have their care tailored to ensure their emotional needs are supported. The findings correspond to international research in this area, and the findings support the need for further research into developing a care pathway that addresses the needs of women following a severe PPH in the short- and long-term.

## Supplementary Material



## Data Availability

The data supporting this research are available from the authors on reasonable request.

## References

[1] Greene RA, McKernan J, Manning E, et al. Major obstetric haemorrhage: Incidence, management and quality of care in Irish maternity units. Eur J Obstet Gynecol Reprod Biol. 2021;257:114-120. doi:10.1016/j.ejogrb.2020.12.021 33383410

[2] Byrne B, Spring A, Barrett N, et al. National Clinical Practice Guideline: Prevention and Management of Primary Postpartum Haemorrhage. National Women and Infants Health Programme and The Institute of Obstetricians and Gynaecologists; 2022. Accessed January 9, 2024. https://www.hse.ie/eng/about/who/acute-hospitals-division/woman-infants/clinical-guidelines/prevention-and-management-of-primary-postpartum-haemorrhage1.pdf

[3] Mavrides E, Allard S, Chandraharan E, et al. Prevention and Management of Postpartum Haemorrhage: Green-top Guideline No. 52. BJOG. 2016;124:e106-e149. doi:10.1111/1471-0528.14178 27981719

[4] Leitao S, Manning E, Corcoran P, et al. SEVERE MATERNAL MORBIDITY in Ireland: Annual Report 2021. National Perinatal Epidemiology Centre; 2023. Accessed January 9, 2024. https://www.ucc.ie/en/media/research/nationalperinatalepidemiologycentre/SMMAnnualReport2021.pdf

[5] Leitao S, Manning E, Corcoran P, et al. Severe Maternal Mortality in Ireland: Annual Report 2020. National Perinatal Epidemiology Centre; 2022. Accessed November 2023. https://www.ucc.ie/en/media/research/nationalperinatalepidemiologycentre/SMMAnnualReport2020.pdf

[6] Parry-Smith W, Okoth K, Subramanian A, et al. Postpartum haemorrhage and risk of mental ill health: A population-based longitudinal study using linked primary and secondary care databases. J Psychiatr Res. 2021;137:419-425. doi:10.1016/j.jpsychires.2021.03.022 33774536

[7] Dunning T, Harris JM, Sandall J. Women and their birth partners’ experiences following a primary postpartum haemorrhage: a qualitative study. BMC Pregnancy Childbirth. 2016;16:80. doi:10.1186/s12884-016-0870-7 27089951 PMC4835830

[8] Thompson JF, Ford JB, Raynes-Greenow CH, Roberts CL, Ellwood DA. Women’s experiences of care and their concerns and needs following a significant primary postpartum haemorrhage. Birth. 2011;38(4):327-335. doi:10.1111/j.1523-536x.2011.00491.x 22112333

[9] Ricbourg A, Gosme C, Gayat E, Ventre C, Barranger E, Mebazaa A. Emotional impact of severe post-partum haemorrhage on women and their partners: an observational, case-matched, prospective, single-centre pilot study. Eur J Obstet Gynecol Reprod Biol. 2015;193:140-143. doi:10.1016/j.ejogrb.2015.07.020 26298809

[10] Rosvig L, Steffensen E, Brogaard L, et al. Women and partners’ experience of major postpartum haemorrhage: a qualitative study. BJOG. 2023;130(9):1087-1095. doi:10.1111/1471-0528.17440 36852514

[11] Carroll M, Daly D, Begley CM. The prevalence of women’s emotional and physical health problems following a postpartum haemorrhage: a systematic review. BMC Pregnancy Childbirth. 2016;16(1):261. doi:10.1186/s12884-016-1054-1 27596720 PMC5011962

[12] Elmir R, Schmied V, Jackson D, Wilkes L. Between life and death: women’s experiences of coming close to death, and surviving a severe postpartum haemorrhage and emergency hysterectomy. Midwifery. 2012;28(2):228-235. doi:10.1016/j.midw.2010.11.008 21251734

[13] Johnson PD, Duzyj CM, Howell EA, Janevic T. Patient and hospital characteristics associated with severe maternal morbidity among postpartum readmissions. J Perinatol. 2019;39(9):1204-1212. doi:10.1038/s41372-019-0426-6 31312037

[14] Briley AL, Silverio SA, Singh C, Sandall J, Bewley S. “It’s like a bus, going downhill, without a driver”: A qualitative study of how postpartum haemorrhage is experienced by women, their birth partners, and healthcare professionals. Women Birth. 2021;34(6):e599-e607. doi:10.1016/j.wombi.2020.12.002 33358131

[15] Nursing and Midwifery Board of Ireland. Practice Standards for Midwives. Nursing and Midwifery Board of Ireland; 2022. Accessed November 2023. https://www.nmbi.ie/NMBI/media/NMBI/NMBI-PracticeStandards-for-Midwives.pdf?ext=.pdf

[16] Ruslin R, Mashuri S, Rasak MSA, Alhabsyi F, Syam H. Semi-structured Interview: A Methodological Reflection on the Development of a Qualitative Research Instrument in Educational Studies Ruslin. IOSR Journal of Research & Method in Education. 2022;12(1):22-29. doi:10.9790/7388-1201052229

[17] Braun V, Clarke V. Thematic analysis. In: Cooper H, Camic PM, Long DL, Panter AT, Rindskopf D, Sher KJ, eds. APA handbook of research methods in psychology, Vol. 2: Research designs: Quantitative, qualitative, neuropsychological, and biological. American Psychological Association; 2012:57-71.

[18] Snowdon C, Elbourne D, Forsey M, Alfirevic Z. Information-hungry and disempowered: a qualitative study of women and their partners’ experiences of severe postpartum haemorrhage. Midwifery. 2012;28(6):791-799. doi:10.1016/j.midw.2011.12.012 22365835

[19] Kramer MS, Berg C, Abenhaim H, et al. Incidence, risk factors, and temporal trends in severe postpartum hemorrhage. Am J Obstet Gynecol. 2013;209(5):449. e1-449.e4497. doi:10.1016/j.ajog.2013.07.007 23871950

[20] Latt SM, Alderdice F, Elkington M, Awng Shar M, Kurinczuk JJ, Rowe R. Primary postpartum haemorrhage and longer-term physical, psychological, and psychosocial health outcomes for women and their partners in high income countries: A mixed-methods systematic review. PLoS One. 2023;18(6):e0274041. doi:10.1371/journal.pone.0274041 37315027 PMC10266652

[21] Turner L, Culliford D, Ball J, Kitson-Reynolds E, Griffiths P. The association between midwifery staffing levels and the experiences of mothers on postnatal wards: Cross sectional analysis of routine data. Women Birth. 2022;35(6):e583-e589. doi:10.1016/j.wombi.2022.02.005 35183474

[22] World Health Organization. WHO recommendations: Intrapartum care for a positive childbirth experience. World Health Organization; 2018. Accessed September 2023. https://iris.who.int/bitstream/handle/10665/260178/9789241550215-eng.pdf?sequence=1 30070803

[23] World Health Organization. WHO recommendations on maternal and newborn care for a positive postnatal experience. World Health Organization; 2022. https://iris.who.int/bitstream/handle/10665/352658/9789240045989-eng.pdf?sequence=1 35467813

