# Supplementary material for: Identifying the needs of women following a severe postpartum hemorrhage
Source: Eur J Midwifery. 2024 Jun 29;8:10.18332/ejm/183027. doi: 10.18332/ejm/183027 (PMC11214268; doi:10.18332/ejm/183027)
Supplement: Supplementary file 1 [file EJM-8-34-s1.pdf]

Attachment 1: Interviews schedule

Part 1 – Welcome

Introduction, name, position.

Thank you for taking part in this research.

Aims of the research.

Reminder that the interview is audiotaped and will be transcribed.

Reminder about confidentiality, anonymity and withdrawal

Do you have any questions about the research?

Part 6- Demographic details

(*AIM: To understand the demography of the women being interviewed, and gain useful information about their childbirth that may not be captured in other parts if this schedule)*

Age

What date and time they gave birth

What gestation they gave birth

Was this their first baby?

If not, how many other children do they have and how old are they?

Was it spontaneous vaginal birth (SVB)/Instrumental birth/ Caesarean section (elective or emergency).

Part 3: Birth story

(AIM: To gain a background into the birth story, capture the recollections of their birth, what was important to them, and what stands out the most)

Can you tell me about your recent birth.

How was the experience for you?

Was there anything that particularly stood out?

Part 4: PPH

(*AIM: To understand the experiences of PPH, how it made them feel at the time, what connotations it brings*)

Were you aware of any complications during your birth?

Were you aware of midwives and/ or doctors concerned about blood loss after your birth?

How/ why?

Can you remember how this made you feel?

Did you feel safe?

Did anybody explain to you what was happening?

Part 4B: Postnatal ward

How did you feel when you were on the postnatal ward?

Did you receive information about your birth experience?

Did you receive any additional information in relation to your blood loss prior to going home from hospital?

Part 5: Looking forward.

(AIM: To understand if this had any positive or negative impact on their birth or recollection, suggestions for improvement)

Did you attend any follow up appointments and if so was this beneficial?

Have you had the chance to talk to anybody in the maternity services about your experience?

When? Who?

If you were to have another baby, what would you like to be different?

What information do think mothers who, like you have had a bleed following the birth, should be given on discharge from hospital?

Part 6: Close

Is there anything else you would like to add?

Thank you for taking the time to participate in this research.

© 2024 Fitzgerald I. et al.
